# Dried human cultured epidermis accelerates wound healing in diabetic mouse skin defect wounds

**DOI:** 10.1038/s41598-022-07156-w

**Published:** 2022-02-24

**Authors:** Michiharu Sakamoto, Takashi Nakano, Itaru Tsuge, Hiroki Yamanaka, Yasuhiro Katayama, Yoshihiro Shimizu, Yoshika Note, Masukazu Inoie, Naoki Morimoto

**Affiliations:** 1 Department of Plastic and Reconstructive Surgery, Graduate School of Medicine, Kyoto University, Shogoin Kawahara-cho 54, Sakyo-ku, Kyoto-City, 606-8507 Japan; 2 Japan Tissue Engineering, Co., Ltd., Gamagori, Japan

**Keywords:** Reconstruction, Experimental models of disease, Preclinical research, Skin diseases, Trauma

## Abstract

Cryopreserved allogeneic cultured epidermis (CE) is used for treating second-degree burn wounds and diabetic foot ulcers; however, the need for cryopreservation limits its use. We have previously reported that CE accelerates wound healing irrespective of its viability and hypothesized that dehydrated CEs lacking living cells may act as an effective wound dressing. We prepared dried CE and investigated its morphological and physical properties and wound-healing effects and compared them with those of cryopreserved CE. Hematoxylin–eosin staining, immunostaining for basement membrane, and electron microscopy revealed that the morphologies of dried CE and cryopreserved CE were comparable and that the membrane structure was not damaged. The breaking strength, modulus of elasticity, and water permeability of dried CE were comparable with those of the cryopreserved CE. Furthermore, the levels of various active cytokines and chemokines in dried CE were comparable with those in cryopreserved CE. Dried CE applied to skin defect in diabetic mice significantly reduced the wound area and increased the new epithelium length 4 and 7 days after implantation, similar to that observed for cryopreserved CE. Consequently, dried CE had similar morphological and physical properties and wound-healing effects compared with those of cryopreserved CE and can be a physiological and versatile wound-dressing.

## Introduction

The shortage of donor sites for skin grafts is a significant problem for the treatment of severe burn injuries. In such cases, cultured epidermal autograft (CEA), which is a cultured keratinocyte sheet prepared from the patient's skin, is a promising treatment option^1^. However, the clinical use of CEA is limited owing to its lower take rate, especially on an infected bed, mechanical fragility, and frequent spontaneous blistering, particularly in the early stages^2^. In addition, a delay of 3 to 4 weeks, required for the preparation of a CEA, creates a fundamental clinical problem, as it occurs during the life-threatening phase in patients with severe burns.

Allogeneic cultured epidermis (allo-CE) is a cultured keratinocyte sheet manufactured from donor cells that can be prepared in advance. In contrast to CEA, allo-CE does not survive on wounds for a long time after application^3^. Instead, allo-CE releases several growth factors that stimulate the activity of recipient cells at the application site, promoting wound healing^3,4^. Allo-CE has been reported to promote wound healing in deep dermal burns^3,5–8^, donor sites^9–12^, and chronic ulcers^13–15^. Cryopreserved allogeneic CE is already commercially available in Korea (Kaloderm, Tego Science, Korea), which is used to treat second-degree burn wounds and diabetic foot ulcers. Similar to Kaloderm, allo-CE is occasionally stored via cryopreservation with cryoprotectant to maintain the morphological characteristics and cell viability; however, maintaining allo-CEs at − 30 °C or lower temperatures in a deep freezer during delivery and storage is challenging.

We have previously reported that CE accelerates wound healing irrespective of cell viability^16^. We prepared four types of CEs with different cell viabilities and applied them to wounds of diabetic mice and observed that CE without any viable cells enhanced epithelialization and wound closure to the same extent as fresh CE even after repeated freeze–thaw cycles. Based on these results, we hypothesized that dried CEs that do not contain living cells can act as an effective wound dressing, which can be stored for a long time at room temperature and can be used immediately off the shelf.

One experimental report and two clinical reports have demonstrated the efficacy of lyophilized CE so far^17–19^, however, detailed investigations regarding the properties of dried CE are lacking and detailed comparison with frozen CE is not available. Therefore, in this study, we investigated the physical properties of dried CE, such as histological properties, tensile strength, and the diversity of cytokines present, and compared its wound healing effect with that of cryopreserved CE in a wound defect model of diabetic mice. Consequently, we elucidated that the properties of dried CE were similar to those of cryopreserved CE and both CEs promoted wound healing to the same extent in a mouse model of skin defect wound.

## Results

### Morphological features of CEs

**Figure 1:**
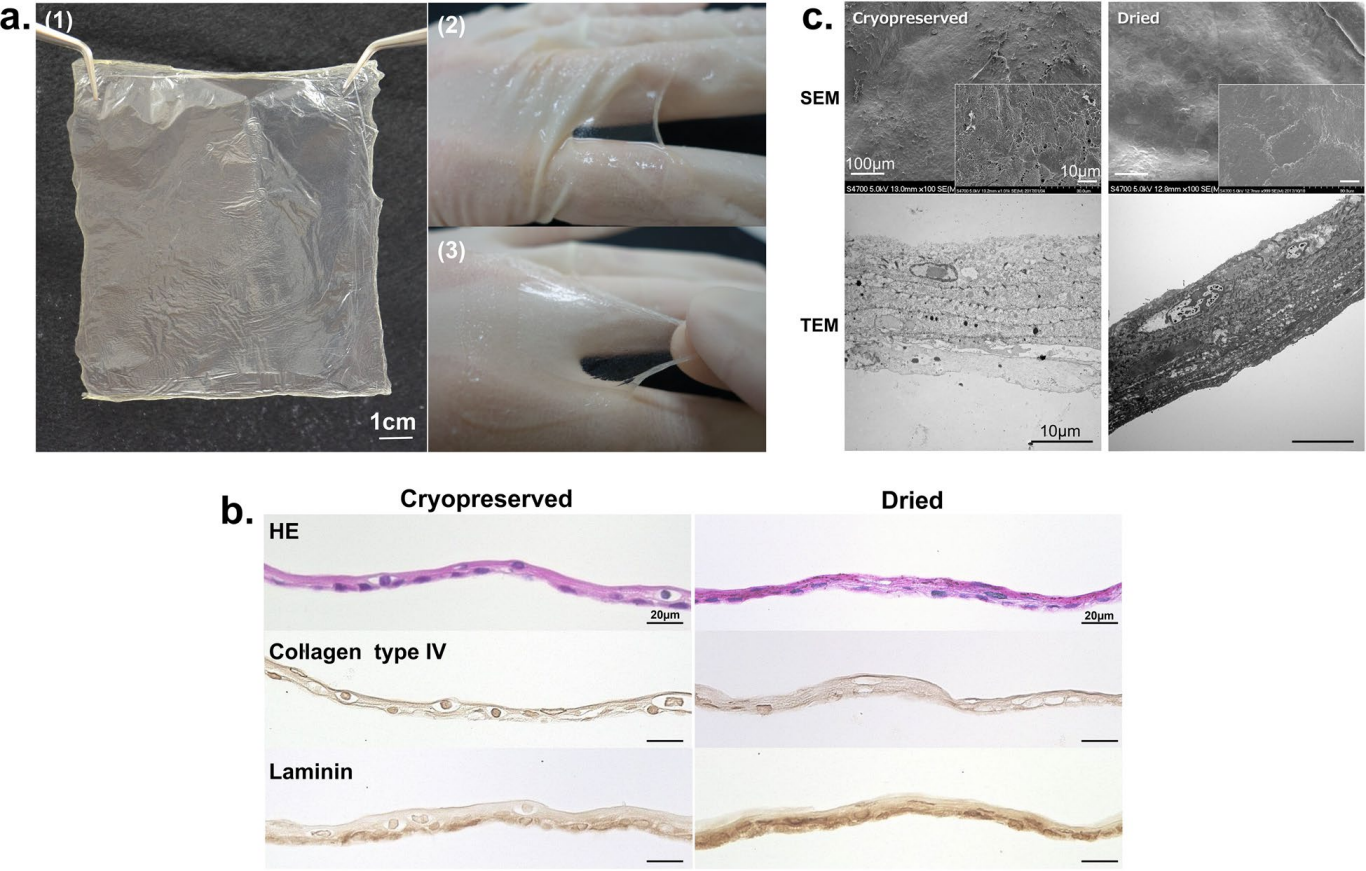
Morphological aspects of a dried CE. (**a**) Dried CE before hydration was cloudy, translucent, and fragile (1). The dried CE transformed into a transparent and flexible sheet with features similar to those of fresh CE sheets after rehydration (2,3). (**b**) Cryopreserved and dried CE sections stained with hematoxylin–eosin and anti-collagen type IV or anti-laminin antibodies. Both types of CEs exhibited similar morphological features, consisting of 4–5 layers of keratinocytes, including a monolayer of basal cells, constituting an undamaged membrane structure. The basal layers of both CEs were detected using immunostaining with an anti-laminin antibody, whereas the collagen type IV staining was not clear. (**c**) SEM and TEM images of cryopreserved and dried CEs. (TEM) Five to six layers of keratinocytes, including the monolayer of basal cell and desmosomal complexes at cell contact points, were observed in both CEs. (SEM) Both CEs appeared similar; the keratinocytes formed a smooth and regular membrane. No crack was observed on the membrane surface. CE, cultured epidermis; SEM, scanning electron microscopy; TEM, transmission electron microscopy.

We prepared CE using Green’s method as described previously^20,21^ with some modifications. The dried CEs were prepared by drying the CEs under controlled temperature and humidity conditions until all the water was completely removed. The dried CE before rehydration was a cloudy, translucent, and fragile sheet. After rehydration, the dried CE transformed into a transparent and flexible sheet with features similar to those of the fresh CE sheet (Fig. 1a). In HE-stained sections (Fig. 1b), both types of CE [Cryopreserved (CP) and Dried (D)] revealed similar morphological features, consisting of 4–5 layers of keratinocytes, including a monolayer of basal cells, constituting an undamaged membrane structure. The morphology of the cells, including the nuclei, was maintained even after the drying and rehydration processes. The basal layers of both CEs were detected using immunostaining with an anti-laminin antibody, whereas the results of collagen type IV staining were not clear.

In TEM studies (Fig. 1c), as observed with HE staining, 5–6 layers of keratinocytes, including the monolayer of basal cells and desmosomal complexes at cell contact points, were observed in both CEs. Both CEs appeared similar in SEM; the keratinocytes formed a smooth membranous structure without irregularities. No cracks were detected on the membrane surface, even in the dried sheets.

### Viability of keratinocytes in the two types of CEs

The cell viability of the two types of CE (CP and D) was investigated by counting viable cells and non-viable cells by the dye exclusion test using trypan blue after trypsinization. The cell viabilities of cryopreserved and dried CEs were 76.4 ± 5.7% and 0 ± 0%, respectively (n = 5, *P* < 0.01). This indicated that all keratinocytes constituting the CE sheet were completely killed by the dehydration procedure and that the dried CEs did not contain any living cells.

### MTT (3-(4,5-dimethylthiazol-2-yl)-2,5-diphenyltetrazolium bromide) assay

The cell viability of CEs was also investigated using the MTT assay. The MTT assay is a measure of the metabolic activity of the cells analyzed; viable cells with active metabolism convert MTT into formazan, while dead cells lose this ability and therefore do not show any signal. Thus, color formation acts as a convenient marker of viable cells. The absorbance at 570 nm was proportional to the number of viable cells. The measured absorbance was as follows: CP: 0.64 ± 0.23; D: 0.07 ± 0.04 (n = 4, *P* < 0.01). This result supported that of the dye exclusion test using trypan blue and confirmed that the dried CE did not contain viable cells.

### Breaking strength and modulus of elasticity

**Figure 2:**
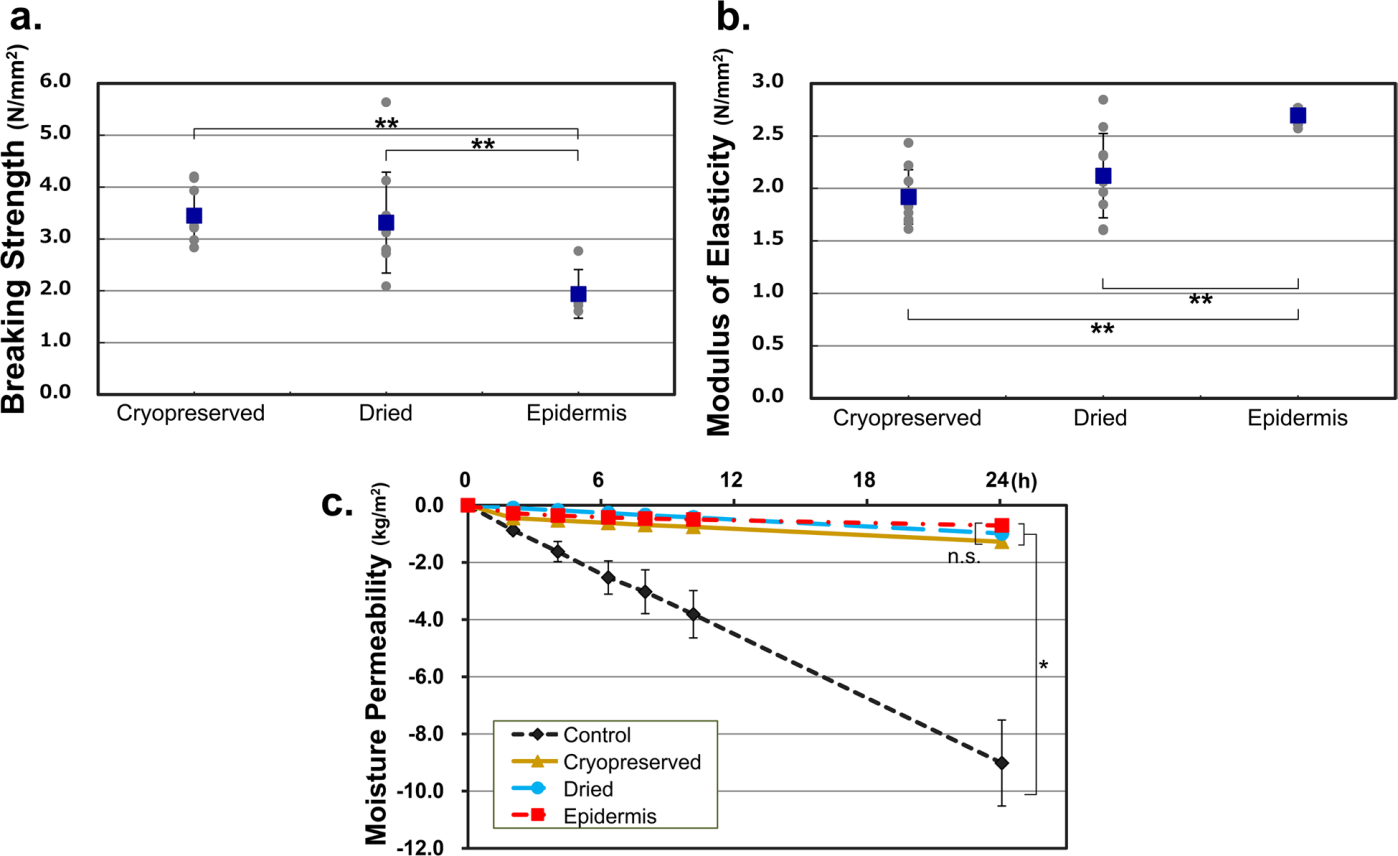
Mechanical properties of a dried CE. (**a**) Breaking strength of cryopreserved CE, dried CE, and epidermis. The breaking strengths of the cryopreserved and dried CEs did not differ significantly (n = 5). (**b**) Modulus of elasticity of cryopreserved CE, dried CE, and epidermis. The modulus of elasticity of the cryopreserved CE and dried CE did not differ significantly (n = 5). (**c**) Moisture permeability of cryopreserved CE, dried CE, and epidermis. A significant difference was not observed between the moisture permeabilities of cryopreserved CE and dried CE (n = 5). The control group represents the evaporation of the saline solution in an uncovered centrifuge tube. CE, cultured epidermis.

Considering the possibility that the drying process can make the CE fragile, we measured the breaking strength and modulus of elasticity of CEs. As a control, an intact epidermis obtained from the donor’s resected skin was used. The breaking strengths of the two types of CEs and the human epidermis are presented in Fig. 2a. The breaking strengths of the two types of CEs were similar and did not differ significantly. The modulus of elasticity of the two types of CEs was similar and did not differ significantly (Fig. 2b). The tensile strength test profile indicated that the dehydration procedure did not interfere with the physical properties of the CEs. Although a significant difference was observed between the two CEs and intact epidermis, it was not substantial, indicating that the man-made epidermis produced by the cell culture technique has physical properties similar to those of the real epidermis.

### Moisture permeability

Since maintaining a moist environment on the wound surface is an important function of a wound dressing, we measured the moisture permeability of the CEs. The moisture permeabilities of CP, D, and the human epidermis were 53.2, 41.0, and 29.6 g/m^2^/h, respectively (n = 5) (Fig. 2c), and CP and D did not differ significantly. Compared to the control group, which revealed natural evaporation from the surface of saline, both CEs provided a moisture-retaining effect.

### Growth factors and cytokines in CEs

**Figure 3:**
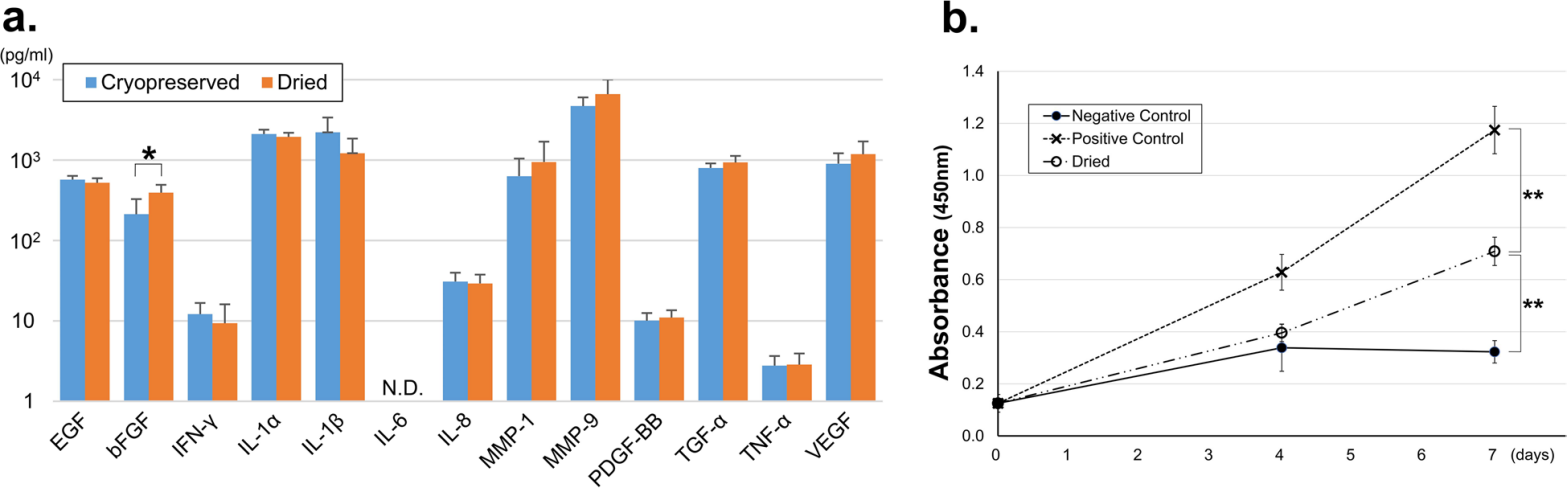
Amount of growth factors in dried CE and enhancement of keratinocyte proliferation by dried CE extracts. (**a**) Amount of growth factors and cytokines in CEs. Except for that in bFGF, the growth factor levels in the two types of CEs did not differ significantly (the amount of bFGF in dried CE was significantly higher than that in cryopreserved CE. * *P* < 0.05). The amount of IL-6 was less than the detection limit, and the amounts of IFN-γ and PDGF-BB were below the lower limit of quantification in both groups. (n = 5). (**b**) Absorbance correlated with keratinocyte proliferation in the WST-8 assay, which is exhibited on the vertical axis. The keratinocytes in the negative control group were cultured in keratinocyte culture medium (EpiLife) only, and a 1% human keratinocyte growth supplement was added to the positive control group. Keratinocyte proliferation was significantly higher on day 7 in the dried CE group than in the negative control group (n = 3, **P < 0.01). CE, cultured epidermis.

The amounts of cytokines and proteases [epidermal growth factor (EGF), basic fibroblast growth factor (bFGF), interferon-gamma (IFN-γ), interleukin (IL)-1a, IL-1b, IL-6, IL-8, matrix metalloproteinase (MMP)-1, MMP-9, platelet-derived growth factor (PDGF)-BB, transforming growth factor (TGF)-a, tumor necrosis factor (TNF)-a, and vascular endothelial growth factor (VEGF)] present in the two types of CEs (CP and D) were assessed using multiplexed sandwich enzyme-linked immunosorbent assay (ELISA) with a Luminex® Assay Human Premixed Multi-Analyte kit (R&D Systems, Inc., Minneapolis, MN). The amounts of growth factors and cytokines are presented in Fig. 3a. Except for that in bFGF, the growth factor levels in the two CEs did not differ significantly. This indicated that the dehydration procedure did not decrease the number of growth factors and cytokines in CEs. IL-6 level was less than the detection limit in both groups.

### Enhancement of keratinocyte proliferation by dried CE extracts

The presence of growth factors in dried CE is not indicative of their biological activity. Hence, we performed a bioassay using keratinocytes to determine whether the growth factors present in dried CE possessed physiological activity. Extracts from dried CE were added to human keratinocytes seeded on a 12-well plate and confirmed to promote keratinocyte growth. Figure 3b demonstrates the ability of dried CE extracts to promote keratinocyte growth. The absorbance at 450 nm in the dried CE group was significantly higher than that in the negative control group on day 7, indicating that the conditioned medium from the dried CE enhanced cell proliferation. (*P* < 0.01).

### Wound healing in full-thickness skin defect model of diabetic mice

#### Wound area

**Figure 4:**
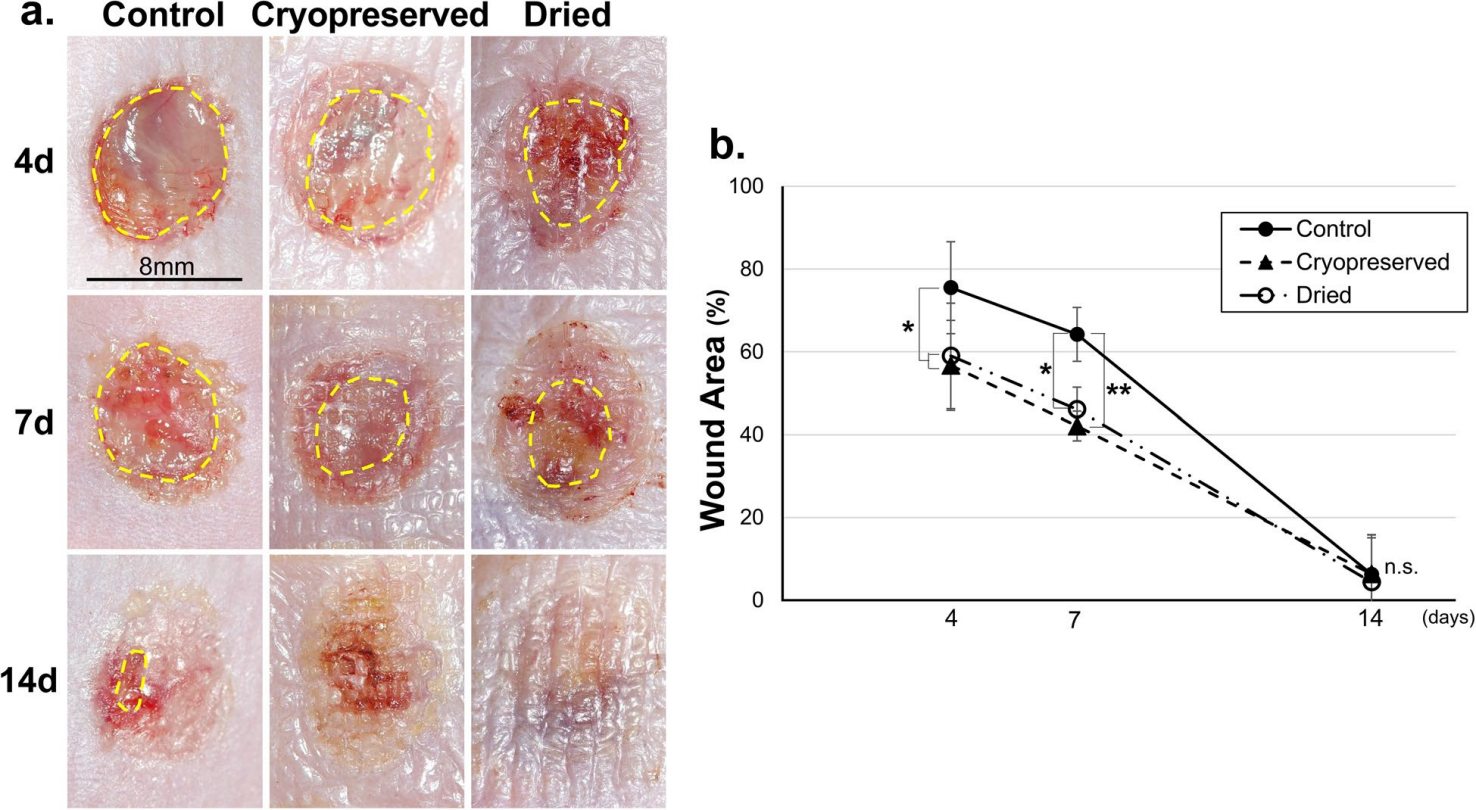
Wounds on the skin defect created on the back of diabetic mice. (**a**) Yellow lines indicate the wound area. In the cryopreserved CE and dried CE groups, no wounds remained on the representative samples on day 14. (**b**) The wound areas in the cryopreserved CE, dried CE, and control groups on days 4, 7, and 14 are depicted. Four and seven days after the operation, the wound areas in the two CE groups were significantly smaller than that in the control group (*P < 0.05, **P < 0.01, n = 6). The wound almost closed in all groups 14 days after the operation. CE, cultured epidermis.

Animal experiments were conducted to compare the wound healing promotion effect of dried CE with cryopreserved CE. The wound area and neoepithelial length were compared on days 4, 7, and 14 after application of dried CE and cryopreserved CE to skin defect wounds created in diabetic mice. The wounds on days 4, 7, and 14 are presented in Fig. 4a. The applied CE was visible as a translucent membrane on the wound surfaces in the two CE groups (CP and D). No obvious infection was detected in any group at any time point. The appearance of wounds between the two CE groups did not differ. The wound area measured from digital photographs is presented in Fig. 4b. Four and seven days after the operation, each of the wound areas in the two CE groups were significantly smaller than that in the control group (*P* < 0.01, control vs. each CE group, n = 6), indicating that both the CEs enhanced wound closure similarly. The wound almost closed in all groups 14 days after the operation, which indicated that CE application did not retard wound healing owing to inflammation or bacterial infection.

#### Length of the neoepithelium

The length of the neoepithelium was determined by measuring the length from the wound edge to the end of the epithelium on HE-stained sections. Besides, day-4 sections were stained with anti-STEM121 antibody to distinguish the transplanted human keratinocytes from the recipient murine epidermis. The anti-STEM121 antibody reacts specifically with the cytoplasmic proteins of human cells and does not cross-react with tissues or extracts from mice or rats. In addition, electron microscopic analysis was performed to confirm the adhesion of the implanted CE to the wound surface.

**Figure 5:**
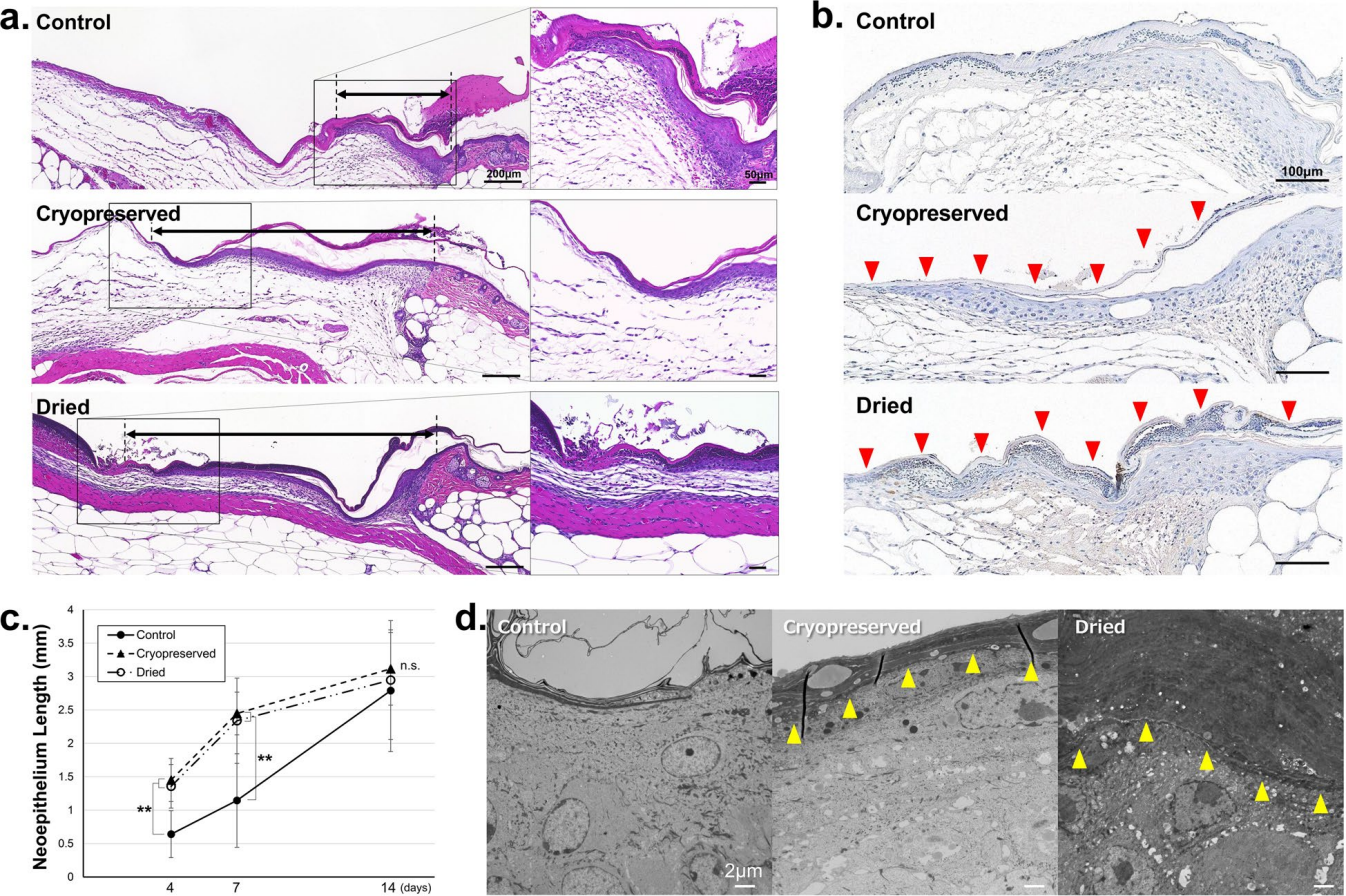
Microscopic images of wound areas in mice of control, cryopreserved CE, and dried CE groups. (**a**) The hematoxylin–eosin stained sections of the wounds in control, cryopreserved CE, and dried CE groups. The arrows indicate the newly formed epithelium. (**b**) Sections stained with anti-STEM121 in the wounds of control, cryopreserved CE, and dried CE groups. The red arrowheads (filled triangle) indicate cultured epidermis. The recipient murine keratinocytes can be easily distinguished from the transplanted CE. (**c**) Length of the epithelium in the wounds in control, cryopreserved CE, and dried CE groups. The epithelial lengths of the two CE groups were significantly higher than those of the control group on days 4 and 7, and significant difference was not observed between the two CE groups. Significant differences between the groups were not observed on day 14. (**P < 0.01, n = 6). (**d**) Transmission electron microscopy (TEM) images of the wound edge in control, cryopreserved CE, and dried CE groups. The wounds were harvested on day 2 and observed using TEM. The yellow arrowheads (filled triangle) indicate the boundary between the implanted CE and recipient keratinocytes. The transplanted human CE and the cell membrane of the mouse keratinocytes completely adhered without any gaps. CE, cryopreserved epidermis.

The HE-stained sections on day 4 are presented in Fig. 5a and sections immunostained with anti-STEM121 antibodies are presented in Fig. 5b. In HE-stained sections, the newly formed epithelium in the control group was short and thick and was composed of multiple layers of keratinocytes. In contrast, the newly formed epithelia in the CE groups were long, thin, and composed of only a few layers of keratinocytes. In the STEM121-stained sections, transplanted human CEs were stained brown with 3,3′-diaminobenzidine-4HCl (DAB); thus, the transplanted CEs could be distinguished from recipient keratinocytes. The transplanted human CE adhered to the wound surface without any gaps, and recipient keratinocytes migrated from the wound edge beneath the CE and constituted an epidermis, after which the applied CE detached from the keratinized surface of the newly formed epidermis in the healed area.

The length of the epithelium is presented in Fig. 5c. The lengths of the epithelium in the two CE groups were significantly more than those of the control group 4 and 7 days after the operation (*P* < 0.01, control vs. CP, control vs. D, n = 6), indicating that both CEs enhanced epithelialization similarly. Significant differences were not observed between the groups.

The TEM images of each group on day 2 are presented in Fig. 5d. In both CE groups, the transplanted human CE and the cell membrane of mouse keratinocytes completely adhered without any gaps.

## Discussion

Many cell-based therapies have been developed so far. However, therapies using cultured autologous cells are tailor-made and time- and cost-exptensive. In contrast, transplantation of allogeneic cells from a donor may be rejected by the recipient’s immune system. Recent studies have presented that the success of cell transplantation as regenerative therapy lies not only in the survival of the cells in the transplanted site but also in the effect of the cytokines and chemokines secreted by the transplanted cells on the recipient cells^22,23^. Based on this idea, cell-derived products, which do not contain viable cells owing to dehydration, have been developed and applied clinically in the field of wound care.

EpiFix™ is a dehydrated human amnion/chorion membrane allograft, which is used to treat diabetic foot ulcers and venous leg ulcers^24^. EpiFix retains soluble biological molecules, such as growth factors, cytokines, and chemokines that cause human dermal fibroblast proliferation and migration of human mesenchymal stem cells (MSCs); it also provides a matrix for cell colonization and acts as a natural biological barrier^25^. A prospective randomized controlled trial for the treatment of diabetic foot ulcers revealed that 77% and 92% of the chronic wounds healed with two-week EpiFix treatment at 4 and 6 weeks, respectively^26^.

OASIS™ is an advanced wound care product comprising a dehydrated extracellular matrix from porcine small intestinal submucosa used to manage wounds. Biologically important components of the extracellular matrix, such as glycosaminoglycans^27^, proteoglycans, fibronectin^28^, and growth factors, such as bFGF^29^ and TGF^30^, are retained in active forms in OASIS. The OASIS was freeze-dried and sterilized with ethylene oxide gas for clinical use. A randomized clinical trial for chronic leg ulcers revealed that compared to 34% in the standard-care group, 55% of the wounds treated with OASIS healed after 12 weeks of treatment^31^. Neither of those contains living cells, and the mechanism of action involves regulatory proteins that enhance wound healing and/or provide a scaffold for cellular invasion and capillary growth, which maintain and support a healing environment for wound management.

We have previously reported that human CE devoid of living cells after repeated freeze–thaw cycles accelerates wound healing and hypothesized that dried CE may be a useful wound dressing, which can be as easy to use as OASIS or EpiFix^16^. Only a few previous reports have described the efficacy of lyophilized CEs. Jang et al. reported that compared to cryopreserved CE in a mouse wound model, human lyophilized CE promoted wound healing^17^. In a randomized control trial where 50 patients with venous leg ulcers were treated with either cryopreserved keratinocytes or lyophilized keratinocytes, the lyophilized cultured epidermal allografts were comparable to the cryopreserved allografts in terms of healing rate, course of healing, relief from the pain, and planimetric changes during healing^18^. In another case report, the chronic leg ulcers of a 67-year-old woman healed within 40 days after treatment with lyophilized cultured epidermal allografts^19^. The authors insisted that this is since wound healing was promoted, as lyophilized CE releases several growth factors and extracellular matrixes that stimulate the activity of the recipient’s cells at the application site. In addition, the membrane structure of CE can contribute to the creation of an environment conducive for recipient cell proliferation and migration in the wound bed. Thus, although few reports revealing the efficacy of lyophilized CE are available, detailed comparisons with cryopreserved CE are lacking. Therefore, we investigated in detail the microstructural changes, physical properties, amount of cytokines present, and in vivo efficacy of CE after dehydration.

The dehydration process is crucial for the preparation of bioactive dehydrated CEs, as harsh chemicals or organic solvents used for dehydration can deteriorate the morphological properties and quantities of preserved growth factors, cytokines, and chemokines. Therefore, we used a simple process to dry the CE without using any chemicals or solvents. Consequently, we were able to fabricate a cell sheet with almost the same strength and flexibility as that of cryopreserved CE, as demonstrated by the results of the strength test and morphological study. Furthermore, we demonstrated that the amount of cytokines in the CEs did not decrease after the drying procedure and that their bioactivity was maintained. Especially, the bFGF promotes the proliferation of fibroblast and endothelial cells and also provokes angiogenesis^32^. Angiogenesis, as an important stage of wound healing, can supply essential nutrients and oxygen during the wound healing process and also promote granulation tissue formation^33^. VEGF is known as a cytokine responsible for the induction of angiogenesis. VEGF promotes cell migration, proliferation, and synthesis of extracellular matrix proteins^34^. The release of these cytokines onto the wound surface actively contributes to wound healing.

The dried CE returned to a flexible membrane structure similar to that of fresh CE only a few minutes after impregnating with saline. This flexibility allowed it to adhere to the wound surface without any gaps at the electron microscopic level, imitating the physiological state in which the wound surface was covered with stratified keratinocytes. This environment accelerated the migration of recipient keratinocytes, promoted epithelialization, and accelerated the reduction of the wound area. The dried CE is advantageous over conventional synthetic wound dressings in these aspects.

This study demonstrated the potential of dried allogeneic CE as a new modality for burn treatment. Although experiments using burn models are most desirable, it was difficult to evaluate wound reduction and epithelialization in burn models in small animals, thus, we conducted in vivo experiments using diabetic mice, according to a previous study^16^. The results of this study can be extrapolated to be of use in burn treatment since healing in second-degree burn wounds was achieved by epithelialization from epithelial progenitor cells remaining in the recipient wound bed. On days 4 and 7, dried CE significantly accelerated epithelialization and wound area reduction. We found that the CE groups healed faster than the control group. The wound area of the CE groups was smaller than that of the control group on day 7, but on day 14, the wounds closed in all groups, and there was no difference in wound area between the control and CE groups. Similarly, the epithelial length did not differ among the groups on day 14. Additionally, since both the CE groups healed on day 14, it is suggested that human CE does not cause abnormal inflammatory reactions or retardations in wound healing. The process of culturing keratinocytes has been reported to decrease the number of Langerhans cells that present T6 and HLA-DR antigens, causing reduced immunogenicity^35^. Clinical studies of allogeneic CE application to burn wounds have demonstrated no prolonged wound healing secondary to immunological rejection^6^.

Thus, similar to OASIS and EpiFix, dried CE may be used as a wound dressing off-the-shelf. As dried CE is composed of keratinocytes, it can be used as a physiological and versatile wound-covering structure. In addition, unlike EpiFix, which is made from perinatal waste, and OASIS, which is made from porcine tissue, dried CE is produced via several passages of culture, owing to which uniform products can be produced in large quantities, stored for a long time at room temperature, and therefore supplied at low manufacturing cost. In addition, they can be used out of the package and can be easily applied immediately without a pre-washing process before use. Sterilization at the end of the manufacturing process reduces the possibility of bacterial contamination and provides the product with an additional level of safety.

The best application of dried CE is for wounds in which hair follicles and other skin appendages remain in the wound, such as second-degree burn wounds and donor sites. For large full-thickness skin defects, dried CE should be used in combination with meshed or patched skin grafts to supply recipient epidermal progenitor cells. Dried CE can be a useful modality for the treatment of severe burns, especially during the first 3 weeks of preparation of the autologous cultured epidermis, which may accelerate the reduction of the wound surface and improve the survival rate and functional prognosis of patients with severe burns.

As a limitation, the superiority of dried CE over several other types of wound dressings already available in clinical practice has not been demonstrated, therefore, further comparative studies are needed. Dried CE is very fragile and delicate, so improvements in the manufacturing process may be necessary to increase its durability in storage. Besides, this study was performed using a xenograft model. Further, we intend to perform a clinical study for assessing the applicability of CEs in clinical situations.

## Conclusions

The properties of dried CE were similar to those of cryopreserved CE, and they contained the same level of biologically active growth factors and cytokines. Both CEs promoted wound healing to the same extent in a mouse model of skin defect wounds. Dried CE can be a physiological and versatile wound-dressing, which can be stored for a long time at room temperature and used immediately off the shelf. As a limitation, this study was performed with a xenograft model using mice. Therefore, a clinical study is necessary. Besides, comparison with the conventional wound dressings and improvement of the usability of dried CE should be addressed in the future.

## Methods

### Ethics statement

All experiments in this study were performed following relevant guidelines and regulations for the use of human tissue samples and animal use.

The human keratinocytes used were obtained from the skin of a supernumerary finger resected from a patient with polydactyly, and the human epidermis used as a control was harvested from the resected skin of a patient who underwent skin resection of her lower extremity at the Kyoto University Hospital. The donor or donor’s parents provided written informed consent before the specimens were obtained. This protocol was approved by the Ethics Committee of Kyoto University Graduate School and Faculty of Medicine (permit numbers R0467-1 and R0690-3).

Our experimental protocol for animal research was approved by the Animal Research Committee of Kyoto University Graduate School of Medicine (permit number Med Kyo 15150), and this study was reported by ARRIVE guidelines. A minimum number of animals was used, and all possible efforts were made to reduce their suffering in compliance with the protocols established by the Animal Research Committee.

### Preparation of cultured epidermis

CE was prepared using Green’s method as described previously^20,21^ with some modifications. The skin without subcutaneous tissue was minced into pieces. After trypsinization, the keratinocytes were isolated from the supernatant, disseminated on an irradiated feeder layer of 3T3-J2 cells in a flask, and cultured in a 3:1 mixture of Dulbecco’s modified Eagle’s medium and Ham’s F12 medium supplemented with fetal bovine serum, insulin, hydrocortisone, cholera toxin, triiodothyronine, epidermal growth factor, and antibiotics in an atmosphere of 10% CO_2_ at 37 °C. Thereafter, the proliferated keratinocytes were collected. To prepare CEs, the keratinocytes were cultivated for approximately one week to confluence. The CE was obtained as keratinocyte sheets, which were detached from the flasks after treatment with dispase II and then backed with non-woven gauze for a carrier of 10 × 8 cm in size for easy handling.

Cryopreserved CEs were prepared by cryopreserving at − 80 °C for more than 1 day. Precisely, CEs were backed with non-woven gauze made of cellulose as a carrier, 10 × 8 cm in size, and then cut into half (5 × 8 cm), put in a cryotube with cryopreservation medium containing 10% DMSO, and cryopreserved at − 80 °C for at least 1 day. The cryopreserved CE was thawed immediately before use by incubating at 37 °C for 8 min in a water bath, followed by gentle washing twice with normal saline solution (NSS) to remove the cryoprotectant.

The dried CEs were prepared by drying the CEs. Precisely, CEs obtained from flasks were transferred into a blank plate and dried under controlled temperature and humidity conditions until all the water was completely removed. The dried CEs quickly recovered the flexible physical properties before drying when they were rehydrated with NSS immediately before use.

### Histological evaluation of CEs

The paraffin-embedded sections of the two types of CEs were stained with hematoxylin–eosin (HE). For immunochemical staining, anti-collagen type IV antibody (ab6586, Abcam) or anti-laminin antibody (ab11575, Abcam) were used as primary antibodies and Histofine Simple Stain Mouse MAX-PO (Nichirei Biosciences Inc.) was used as the secondary antibody. Further, the samples were exposed to DAB. The specimens were then observed under an optical microscope.

### Electron microscopic analysis of CEs

The CEs were fixed overnight in 0.1 M phosphate buffer with 2% glutaraldehyde and 4% paraformaldehyde at 4 °C. After fixation with 1% OsO4 for 2 h, the samples were dehydrated and dried. For SEM, the samples were coated with a thin layer of platinum palladium. For TEM, thin sections were stained with 2% saturated uranyl acetate solution and 2.5% lead citrate solution.

### Cell viability of keratinocytes in CE

#### Viable cell count

Each CE was trypsinized and mixed with 0.4 w/v % trypan blue. The numbers of viable and non-viable cells were counted under a phase-contrast microscope. The cell viability (the number of viable cells/the number of all cells) of each CE was calculated.

#### MTT assay

Small samples (8 mm in diameter) were prepared from each CE (n = 4). Each sample was placed in wells of a 24-well plate, which contained MTT solution, and the absorbance at 570 nm (with a reference wavelength of 650 nm) of the reacted reagent was measured using a microplate reader.

#### Tensile strength test

As a control, an intact epidermis was obtained from the donor’s resected skin after dispase treatment. Five pieces of CE (5 × 50 mm) were prepared from each CE. The thickness of the CE pieces and epidermis was measured using a digital measuring instrument. Both ends of a CE piece or epidermis were tensioned until breakage at a speed of 30 mm/min. The modulus of elasticity was calculated from the tension-strain curve at a strain ratio of 10–20%. The breaking strength is the tensile strength at break.

#### Moisture permeability

The top opening of a 15 mL centrifuge tube containing 13 mL saline was covered with each sample and sealed with adhesive. As a control, a tube without any cover (that was left open) was prepared. The tubes were placed at 25 °C in an atmosphere of 50% relative humidity, and their weights were measured after 24 h.

#### Cytokine assay

Five sheets (8 × 10 cm) in each group (CP and D) were prepared. Each sheet of CE was incubated in a centrifuge tube containing 4 mL buffer [10× RIPA buffer: 1% Triton X-100: distilled water: 1:1:8] at 4 °C for 24 h. Further, 140 µL supernatant from each tube was collected and the amount of each factor in the lysis buffer was measured according to the manufacturer's protocol. The sample from each well was imaged with Bio-Plex 200 system (Bio-Rad Laboratories, Inc.) using a set of filters to differentiate excitation levels and analyzed using Bio-Plex Manager software version 6.1. In this measurement, the value for IL-1a exceeded the limit of measurement and was not reliable; hence, we used the value that was remeasured using Invitrogen’s ELISA kit (R&D Systems Inc.).

### Enhancement of keratinocyte proliferation by dried CE extracts

#### Preparation of a conditioned medium from a dried CE

A sheet of dried CE (8 × 10 cm) was incubated in a 50 mL centrifuge tube containing a 20 mL serum-free medium (EpiLife; MEPI500CA) at 4 °C for 24 h. The supernatant was then used as the conditioned medium.

#### Keratinocyte seeding

Human keratinocytes were used at passage 5. In total, 2.0 × 10^4^ keratinocytes in 1 mL of EpiLife supplemented with 1% human keratinocyte growth supplement (HKGS)(S0015; ThermoFisher Scientific) were seeded on each well of a 12-well plate (n = 24) and incubated at 37 °C in the presence of 5% CO_2_ for 24 h. After incubation, cell numbers at day 0 (D0) were evaluated using the WST-8 assay. The other 18 samples were allocated to the three groups [negative control (NC), positive control (PC), and dried] and further cultured.

#### Cell culture

The medium was changed to the medium required for each group (mentioned below) after washing twice with PBS and incubated at 37 °C in the presence of 5% CO_2_.

NC (negative control): 1 mL EpiLife without HKGS.

PC (positive control): 1 mL EpiLife with 1% HKGS.

Dried: 900 µL EpiLife without HKGS and 100 µL conditioned medium from dried CE.

After 4 days (D4) and 7 days (D7), cell numbers were evaluated using the WST-8 assay (n = 3, at each time point).

### Application of CEs on the skin defects of diabetic mice

The animal study was performed according to a previous study^16^. Diabetic mouse (BKS.Cg-Dock 7 m + / + Leprdb/Jcl) used in this study is a mutant strain that develops marked diabetic symptoms spontaneously, including obesity, overeating, and hyperinsulinemia, and is widely used in diabetes research or studies on wound-healing^36,37^. Thirty mice (10-week-old male) were used and allocated to three groups (control, CP, and D). Two symmetrical full-thickness skin defects 8 mm in diameter were created on both sides of the dorsum of each mouse. Each wound was covered with the respective CEs (1.5 × 1.5 cm) in the CP and D groups. In all groups, including the control group, the wounds were covered with a non-adherent flexible contact layer (UrgoTul) and polyethylene films containing absorbent cotton (Derma-Aid) to maintain the wounds in a moist environment. One mouse in each group was sacrificed on day 2, and the wounds were harvested for TEM.

On days 4, 7, and 14, three mice from each group were sacrificed and digital photographs were captured to evaluate the size of the wound area. The wounds were then harvested and used for HE and immunohistochemical staining. Day-4 sections were stained with anti-STEM121 antibody to distinguish the transplanted human keratinocytes from the recipient murine epidermis. For STEM121 staining, anti-STEM121 mouse monoclonal antibodies (dilution 1:1,500, code Y40410 Cellartis, Takara Bio Inc.) and Histofine Simple Stain Mouse MAX-PO (Nichirei Biosciences Inc.) were used. The samples were subsequently exposed to DAB and counterstained with hematoxylin.

### Assessment of the wound area and epithelialization

The wound area was measured using ImageJ version 1.45 and is presented as a percentage relative to the original wound area. The length of the neoepithelium was determined by measuring the length from the wound edge to the end of the epithelium on HE-stained sections using an optical microscope.

### Statistical analysis

Statistical significance was determined using the t-test for comparisons between two groups and one-way analysis of variance with the Tukey–Kramer post-test multiple comparisons for three or more groups after normality was confirmed by the Shapiro–Wilk test. All data are expressed as mean ± standard deviation. Statistical significance was set at *P* < 0.05.
